# The expression of hyperpolarization activated cyclic nucleotide gated (HCN) channels in the rat ovary are dependent on the type of cell and the reproductive age of the animal: a laboratory investigation

**DOI:** 10.1186/1477-7827-6-35

**Published:** 2008-08-18

**Authors:** John Yeh, Beom Su Kim, Larry Gaines, Jennifer Peresie, Carly Page, Armando Arroyo

**Affiliations:** 1 Department of Gynecology-Obstetrics, University at Buffalo, The State University of New York, Buffalo, New York, 14222, USA

## Abstract

**Background:**

Aim of this study was to test the hypothesis that levels of hyperpolarization activated cyclic nucleotide gated channels 1 to 4 (HCN1-4) are linked to the reproductive age of the ovary.

**Methods:**

Young, adult, and reproductively aged ovaries were collected from Sprague-Dawley rats. RT-PCR and western blot analysis of ovaries was performed to investigate the presence of mRNA and total protein for HCN1-4. Immunohistochemistry with semiquantitative H score analysis was performed using whole ovarian histologic sections.

**Results:**

RT-PCR analysis showed the presence of mRNA for HCN1-4. Western blot analysis revealed HCN1-3 proteins in all ages of ovarian tissues. Immunohistochemistry with H score analysis demonstrated distinct age-related changes in patterns of HCN1-3 in the oocytes, granulosa cells, theca cells, and corpora lutea. HCN4 was present only in the oocytes, with declining levels during the reproduction lifespan.

**Conclusion:**

The evidence presented here demonstrates cell-type and developmental age patterns of HCN1-4 channel expression in rat ovaries. Based on this, we hypothesize that HCN channels have functional significance in rat ovaries and may have changing roles in reproductive aging.

## Background

Molecular studies of ovarian granulosa cells have determined that the granulosa cells of various species express potassium, calcium, sodium, and chloride channels. These channels have electrical activity and generate action potentials. Porcine granulosa cells express a potassium current (I_A_), a delayed rectifier K^+ ^current (I_K_) and Ca^2+ ^currents [1,2]. Ion channels such as Kv1.1, Kv1.2, Kv1.3, Kv1.4, Kv1.5, Kv1.6, KCNQ1, KCNE1 have been identified in porcine granulosa cells [2]. Kir6.1 and Kv4.2 are expressed in human granulosa cells [3,4]. Ca^2+ ^subunits Cav1.2 and Cav3.2 are expressed in human granulosa cells and calcium type currents are also found in human granulosa cells [5,6]. Human granulosa cells express a Ca^2+ ^activated K^+ ^current (BK_Ca_), a transient outward K^+ ^current and an ATP-sensitive potassium channel [3,4,7]. In hen granulosa cells, chloride channels are activated by cAMP during LH-stimulated progesterone production [8].

During aging, potassium, calcium and sodium channels activities and levels are altered. For the potassium and calcium channels, the channels in the cells in the brain, heart, liver, and pancreas all change during the process of aging [9-12]. Cumulatively, these changes include a decrease in the total number of ion channels present and alterations in the distribution and activity of the channels. For the sodium channels, the changes associated with developmental aging in retinal ganglion cells, myocardium and in kidney epithelium cells include shifts in the number and alterations in conduction activity [13-15]. These reports suggest that there are specific age-related patterns in the expression and physiological activity of ion channels.

Hyperpolarization activated cyclic nucleotide gated (HCN) channels generate a pacemaker current (I_h_) that controls spontaneous pacemaker activity in the heart and brain [16-19]. There are four members of the HCN gene family and they belong to the voltage-gated K+ superfamily. The four forms of HCN genes (HCN1-4) have highly conserved core transmembrane and cyclic nucleotide binding regions, with each of the four proteins having a six transmembrane region. The four HCN genes have different distributions in the heart and brain, suggesting that they have different functions. HCN channels have been in found in neurosecretory neurons of the hypothalamus, retinal rod photoreceptors, hair cells of the auditory system, olfactory neurons, spinal cord dorsal root ganglion neurons, and the enteric nervous system [16-25]. The wide distribution of the HCN channels suggests that they have roles in a number of different physiological conditions. In addition to the wide distribution of these channels, it has been previously reported that HCN4 expression in the hippocampus is related to developmental age, suggesting that these channels also have aging-related changes [23,24].

To our knowledge, no prior studies have investigated the HCN channels in the ovary. Given the important roles of HCN in other organs and given the aging-related changes found in potassium, calcium, sodium and HCN channels, it was hypothesized that HCN channels play vital roles in the ovary and that alterations of their expression would be found during reproductive aging. In this study, we analyzed the expression and localization of HCN1-4 in the rat ovary to assess this postulate.

## Methods

### Animals and treatment

Sprague-Dawley rats (Harlan, Indianapolis, IN) of three age groups were studied: 1.) "young", 26 days old, immature control females; 2.) "adult", 65–75 day old, adult control females and; 3.) "reproductive aging", 8–9 month old retired breeders, experimental females with declining fertility [26]. The animals were maintained under standard housing conditions with a 12 h:12 h light cycle. They were provided access to standard rat chow (Harlan, Indianapolis, IN) and water ad libitum. The animals were euthanized by an overdose of carbon dioxide. Subsequently, both ovaries were dissected out from each animal; one ovary was snap frozen and stored at -80°C while the other one was fixed in 10% formalin and stored at 4°C for paraffin sectioning. All procedures were approved by the Institutional Animal Care and Use Committee of the University at Buffalo (GYN07042N).

### RNA isolation and RT-PCR

Rat ovarian total RNA was isolated using Trizol (GibcoBRL, Life Technologies, Grand Island, NY). RT-PCR was performed as previously described by our laboratory [25], using a Promega Access RT-PCR kit (Access RT-PCR System, Promega, Madison, WI). PCR primers were designed to amplify rat HCN1-4 mRNA (Table 1) and were slightly modified from mouse primers used previously [25]. Positive control for HCN1-4 was brain RNA and the negative control was running the PCR reaction without the cDNA template. PCR conditions were as follows: 45°C for 45 min, 94°C for 2 min, and then 40 cycles of 94°C for 30 sec, 60°C for 1 min, 68°C for 2 min, and a final extension of one cycle at 68°C for 7 min. The analysis of the RT-PCR reaction products was by agarose gel electrophoresis.

**Table 1:** Primers for RT-PCR reactions

Gene	GenBank Accession No.	Forward primer (5'-3')	Reverse primer (3'-5')	Size of product (bp)
HCN1	NM_053375	TTCATGCAGAGGCAGTTCAC	CACGGTGTTGTTGTTTGCTC	248
HCN2	NM_053684	CCATGCTGACAAAGCTCAAA	CGAGCTGAGATCATGCTGAA	377
HCN3	NM_053685	TCGGACACTTTCTTCCTGCT	GGTTGAAGATGCGAACCACT	364
HCN4	NM_021658	GGGCTTCTCCTGTAGCCTTT	TGAGCTTCAGGTCCTGTGTG	219

### Western blotting

Total protein was isolated by procedures used previously [25,26]. In brief, rat tissues were lysed in RIPA buffer, containing 50 mM Tris-HCl, 150 mM NaCl, 0.1% sodium dodecyl sulfate (SDS), 1% NP-40, 1 mM phenylmethanesulfonyl fluoride (PMSF), and 0.5% N, N'-dicyclohexylcabodiimide (DCC) [25,26], along with protease inhibitors (Sigma, St. Louis, MO, 1:100). The protein concentrations were determined by the Bradford method (Bio-Rad, Hercules, CA). Fifty micrograms of protein from rat tissues under reducing conditions were loaded onto a 10% (HCN2 and HCN3) or an 8% (HCN1 and HCN4) Tris-Glycine SDS-polyacrylamide gel (Invitrogen, Carlsbad, CA). After electrophoresis, the proteins were electrically transferred to a nitrocellulose membrane (VWR International, West Chester, PA), blocked with 5% skim milk in TTBS (TBS with 0.1% Tween 20), and then incubated overnight at 4°C with rabbit polyclonal antibodies against HCN1-4 (anti-HCN 1, 2, 3 or 4; product # APC-056, APC-030, APC-057, APC-052, respectively; Alomone Labs Ltd., Jerusalem, Israel) [26] at a dilution of 1:200. Horseradish peroxidase (HRP)-conjugated goat anti-rabbit IgG (1:2500; Amersham Pharmacia, Piscataway, NJ) was used to identify the protein bands and they were amplified using SuperSignal West Pico Chemiluminescent Substrate Kit (Pierce Biotechnology, Rockford, IL). Visualization of the protein bands was by CL-Xposure film (Pierce Biotechnology).

### Immunohistochemistry and H score semiquantitative analysis

After fixation, ovaries were embedded in paraffin and cut at 4 μm thick sections that were placed on Starfrost Adhesive positively charged microscope slides (Mercedes Medical, Germany) and the procedures used were as previously described [27]. Sections were deparaffinized using xylene and rehydrated using graded alcohol series. Sections were rinsed in distilled water then incubated for 30 minutes in 4N HCl at 37°C for antigen retrieval. Slides were cooled to room temperature, and then washed in PBS for 5 minutes. The slides were then placed in sodium borohydride diluted in PBS at a concentration of 1 mg/ml. The slides were then rinsed three times in PBS. The tissue sections were then blocked for 1 hour at room temperature using 5% goat serum and 5% BSA. The slides were transferred to a humidified chamber, and a rabbit polyclonal primary antibody against one of the HCN channels described in Western blotting. The HCN2 and HCN3 anitibodies were applied at dilutions of 1:200, and HCN1 and HCN4 were at dilutions of 1:50. As negative control, the primary antibodies were omitted. To confirm specificity of immunostaining, an additional negative control was performed for each of the channels. The anti-HCN1-4 antibodies were pre-incubated with the appropriate antibody control antigen as follows: HCN 1 _6–24 _Peptide, HCN 2 _147–161 _Peptide, HCN 3 _727–744 _Peptide, and HCN4-GST fusion protein (provided by Alomone labs). For the HCN1-3 preadsorption control solution, 1 μg of peptide was incubated with 1 μg of antibody and for HCN 4 preadsorption control solution, 3 μg of fusion protein was incubated with 1 μg of antibody for one hour at 37°C, centrifuged at 12,000 rpm, and then applied to the tissue sections. The slides were incubated with primary antibodies overnight at 4°C. The following morning, slides were washed 3 times in PBS for 5 minutes each. To visualize the primary antibody, Alexa Fluor goat anti-rabbit 594 (Molecular Probes, Eugene, OR) were applied to the tissue sections for 30 minutes at room temperature in the dark. To visualize HCN1, 2, and 3, the Alexa Fluor was applied at a dilution of 1:1000. To visualize HCN4, the secondary antibody was applied at a dilution of 1:500. Slides were air dried for 1 hour and cover-slipped using ProLong Antifade (Molecular Probes). The sections were then viewed on a Nikon Eclipse E400 fluorescent Microscope (Micro Video Instruments, Avon, MA) using the appropriate filters.

A modified H score system was used to analyze the HCN channels 1 to 4 staining [27,28]. This scoring system was based on two criteria: the distribution of the staining, and the intensity of the staining. The following scale was used to determine the distribution of the stain in a structure: less than 50% of the structure stained was scored as 1, greater than or equal to 50% of the structure stained was scored as 2. To determine the intensity of the stain the following scale was used: no stain = 0, weak staining = 1, moderate staining = 2, and intense staining = 3. The numbers obtained for the distribution and intensity were then multiplied together for a combined score. A total score of 6 was the maximum for any structure. Two independent observers scored the sections for H score analysis. The results from each reviewer were compared and any discrepancy greater than 10% resulted in a reevaluation with both reviewers. Follicles were divided into classes based on the criteria of Oktay et al [29]. Primordial, primary, preantral, and antral follicles were included in the H score analysis, as well as thecal cells, oocytes, and corpora lutea.

### Statistical analysis

Three separate rats in each age group were used for the RT-PCR and western blot experiments. Data for the ovarian immunofluorescence H score analysis are presented as mean +/- standard error and represent results from experiments repeated in triplicate. Statistical analyses of the ovarian H scores for HCN1-4 were performed using a one-way ANOVA followed by a linear contrast (SPSS version 11). P < 0.05 was considered statistically significant.

## Results

### Gene expression and western blot analysis of HCN1-4

**Figure 1:**
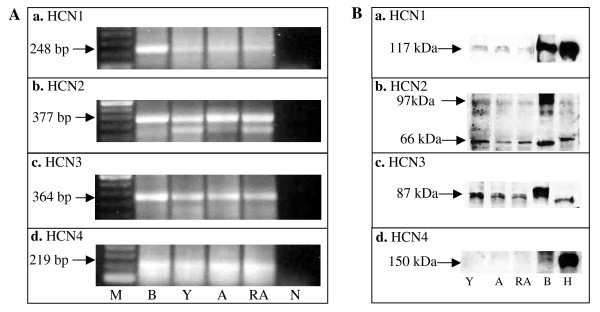
**(A) **RT-PCR analysis of ovarian HCN1-4 gene expression. **(a) **A 248 bp HCN1 RT-PCR band in all three developmental ages studied. **(b) **A 377 bp HCN2 RT-PCR band was evident in all three developmental ages studied. **(c) **A 364 bp HCN3 RT-PCR band was expressed in all three developmental stages studied. **(d) **A 219 HCN4 RT-PCR band was present in all three developmental stages studied. n = 3 animals studied per gene per reproductive stage. M = marker; B = brain; Y = young; A = adult; RA = reproductive aging; N = negative; H = heart. **B**. Western blot analysis of ovarian lysates for HCN1-4 protein expression of young, adult and reproductively aged rats. **(a) **Western blot analysis for HCN1 in ovaries. **(b) **Western blot analysis for HCN2 in ovaries. **(c) **Western blot analysis for HCN3 in ovaries. **(d) **Western blot analysis for HCN4 in ovaries. n = 3 animals studied per protein per reproductive stage.

HCN1-4 mRNA expression in the rat ovary was determined by RT-PCR (Figure 1A). For all four HCN channels, RT-PCR demonstrated the presence of these mRNA in the ovaries in all reproductive stages studied. Western blot analysis showed protein bands for HCN1-3 in rat ovaries of the three reproductive stages studied (Figure 1B). For HCN4, no protein was detected in the rat ovarian tissue by our whole ovarian extract western blot analysis. However, a 150 kDa protein band was found for rat brain and heart, two positive controls.

### Immunohistochemistry of ovarian HCN1-4

**Figure 2:**
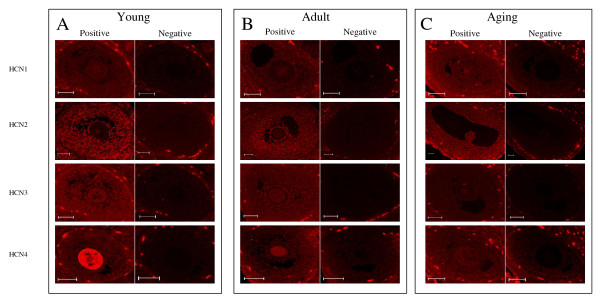
Immunofluorescence localization of HCN1-4 in young, adult and reproductively aged rat ovaries. Experimental and negative control studies are presented from consecutive ovarian sections for each HCN protein for each reproductive state. **(A) **Localization of HCN1-4 channels in the young ovary. **(B) **Localization of HCN1-4 channels in the adult ovary. **(C) **Localization of HCN1-4 channels in the reproductive aging ovary. n = 3–9 animals studied per protein per reproductive stage.

Figure 2 depicts ovarian follicles and the staining patterns found using immunofluorescence to analyze for the localization of HCN1-4 in young, adult and reproductively aged rat ovaries. By H score analysis (Figure 2), differences were detected in the spatial and temporal localization of HCN1-4 in the rat ovary. All four HCN proteins were detected in ovaries of all three reproductive ages. However, there were specific spatial protein differences in the distribution of HCN1-4. For HCN1, HCN2, and HCN3, the proteins were found in oocytes and in the granulosa cells of primary, preantral, and antral follicles. In addition, all three proteins were found in thecal cells. Furthermore, all three proteins were localized to corpora lutea. For HCN4 experimental tissue sections, HCN4 protein expression was localized only to the oocytes. In the HCN1-4 negative control experiments, both types of negative control experiments, the preabsorbed control experiments and the omission of the primary antibody experiments, were appropriately negative.

**Figure 3:**
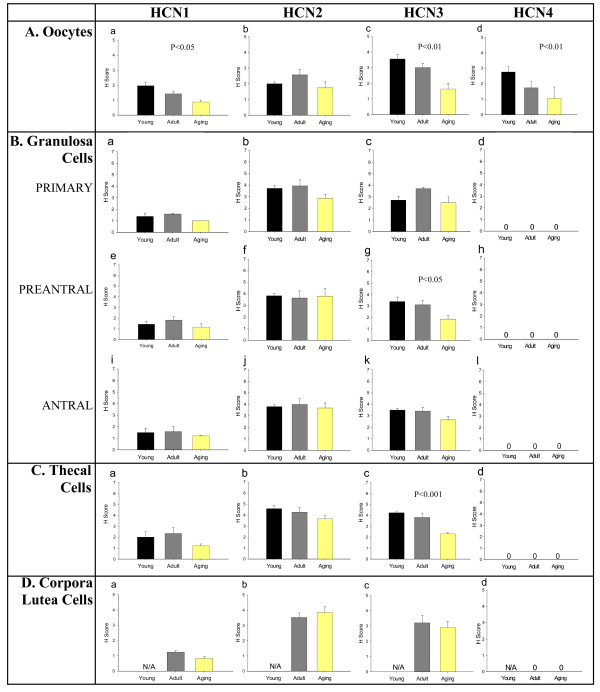
H Score analysis of HCN1-4 channels in the different ovarian structures throughout the aging process. **(A) **The H scores of HCN1 (a), HCN2 (b), HCN3 (c), and HCN4 (d) channels in the oocytes during the reproductive aging process. **(B) **H score analysis of granulosa cells in different follicle classes. (a-d) H scores of HCN1-4 channels in primary follicles. (e-h) Results of H score analysis of HCN1-4 channels in preantral follicle granulosa cells. (i-l) H scores for HCN1-4 channels in the granulosa cells of antral follicles. **(C) **H score analysis of HCN1 (a), HCN2 (b), HCN3 (c), and HCN4 (d) expression in theca cells. **(D) **H score analysis of HCN1-4 channels (a-d respectively) in the corpora lutea. N/A – not applicable, as the young rats do not yet have corpora lutea. n = 3–9 animals studied per protein per reproductive stage.

In addition to the spatial findings above, there were age-related findings related to the reproductive age of the ovaries for the specific ovarian structures studied (Figure 3). For the following structures, there were differences in the H scores for the HCN proteins for the three reproductive ages studied: 1.) oocytes: HCN1 (decline in H score with increasing reproductive age; p < 0.05), HCN3 (decline in H score with increasing reproductive age; p < 0.01) and HCN4 (decline in H score with increasing reproductive age; p < 0.01); 2.) preantral follicle granulosa cells: HCN3 (decline in H score with increasing reproductive age; p < 0.05); 3.) primary follicle granulosa cells: HCN3 (decline in H score with increasing reproductive age; p < 0.01); 4.) thecal cells: HCN3 (decline in H score with increasing reproductive age; p < 0.01).

## Discussion

All four types of HCN channels are expressed in the ovary as evidenced by RT-PCR, western blot, and immunohistochemical results presented in this report. To our knowledge, this is the first description of the changes in distribution of the HCN channels in ovarian structures in the reproductive life-cycle. These channels have specific patterns in the different ovarian cell types. HCN channels 1–3 are expressed in oocytes, granulosa cells of primary, preantral, and antral follicles, the thecal cells, and in luteal cells, while HCN4 is only expressed in oocytes. This suggests that different ovarian structures use different combinations of HCN channels for normal physiological function. Furthermore, HCN4 appears to be oocyte specific and, thus, this protein may be useful to define the physiological status of an oocyte.

Ion channels are involved in ovarian steroidogenesis. Potassium channels mediate gonadotropin regulated progesterone secretion in human granulosa cells [3,4,7,30]. L- and T-type Ca^2+ ^channels mediate hCG stimulated progesterone secretion in human granulosa cells [5]. Sodium channels down regulate progesterone production in primate granulosa cells [31]. Potassium channels and cAMP are involved in FSH-stimulated progesterone production in pig granulosa cells [32]. Given that HCN channels are located in all the cell types which are involved in steroidogenesis, the granulosa, theca and corpora lutea cells, it would not be unreasonable to hypothesize that the HCN channels also participate in this important ovarian activity. HCN channels have been identified in secretory cells including GnRH neurons, pancreatic β-cells, and pituitary lactotrophs [16-19,25]. Several studies have described membrane hyperpolarization in granulosa cells. Activation of BK_Ca _channels resulting in membrane hyperpolarization is required for steroidogenesis in human luteinized granulosa cells [7]. Thus, membrane hyperpolarization may be a mechanism controlling steroid production in the granulosa cells of the ovary. In contrast to most voltage gated channels, HCN channels are activated by membrane hyperpolarization [16-19]. In granulosa cells it is possible that hyperpolarization of the cell membrane could activate HCN channels, thereby resulting in membrane depolarization. Depolarization could activate calcium and cAMP signaling, thus resulting in activation of steroidogenic enzymes and thereby increasing steroid production. This hypothesis would be supported by demonstration of functional HCN channels in granulosa cells.

There are age related changes in potassium, calcium and sodium channel expression [9-15]. In the data presented in this report, HCN1 and HCN2 had minimal variation through the aging process, with HCN1 only exhibiting declining levels in the oocytes during reproductive aging. This suggests that the expression of these two channels remains relatively constant throughout granulosa and thecal cell reproductive aging and may have minor or unchanging roles in ovarian physiologic functions such as steroidogenesis or peptide hormone production. HCN3 has different expression patterns in the granulosa cells and theca cells during the aging process, indicating that there is an age-dependent expression of HCN3 in ovarian structures, suggesting that the changes in steroidogenesis during aging might be modulated through this protein. HCN3 expression decreases during aging in oocytes, granulosa cells of preantral follicles, and in theca cells, suggesting a possible function in the decrease of ovarian function in advancing reproductive age. The mechanism of this decline is not yet known and understanding of the mechanism could lead to further insights into the overall aging-related reduction of ovarian function. To date, the physiologic processes in reproductive aging are not yet fully understood. HCN channels may play a role in ovarian aging and, in addition, they could serve as immunohistochemical biomarkers for reproductive aging.

HCN4 is an oocyte specific channel and may be an indicator of oocyte quality. There is a linear decrease in the expression of HCN4 throughout the reproductive aging process in the female rat. Brewster et al. and Surges et al. showed a steady decrease in HCN4 expression throughout the maturation process of the rat hippocampus [23,24]. In the ovary, growth differentiation factor 9 (GDF-9) has been found to be oocyte specific [33-35]. GDF-9 has been demonstrated to be essential to the growth and differentiation of early ovarian follicles. In cultured bovine granulosa cells, GDF-9 stimulated the proliferation of granulosa cells from small and large antral follicles and can disrupt the production of progesterone and estradiol [36]. The functions of HCN4 in the oocyte have not been determined to date, but it is possible that the channel may also be necessary for growth and differentiation of ovarian follicles or for steroid production.

## Conclusion

In conclusion, HCN1-4 channels are expressed in the ovary and there are differential expression patterns for the channels. HCN1-3 are expressed in ovarian structures including oocytes, granulosa and thecal cells, and in luteal cells, while HCN 4 is only expressed in the oocytes. There are decreases in the expression of HCN1, 3, and 4 in the oocytes during reproductive aging, with the decrease in HCN4 being the most pronounced. HCN3 expression are also decreased in the granulosa cells of preantral follicles and theca cells in reproductive aging. Future studies need to be conducted to determine the specific roles of HCN channels in the ovaries and the physiological reasons for the changes in the expression of the channels through the aging process.

## Abbreviations

HCN: hyperpolarization activated cyclic nucleotide gated channel; RT-PCR: reverse transcriptase polymerase chain reaction; mRNA: messenger ribonucleic acid; Kv1.1 – 1.6: potassium voltage-gated channel, shaker-related subfamily, members 1–6; KCNQ1: potassium voltage-gated channel, subfamily Q, member 1; KCNE1: potassium voltage-gated channel, Isk-related family, member 1; Kir6.1: potassium inwardly-rectifying channel, subfamily J, member 8; Kv4.2: potassium voltage-gated channel, Shal-related family, member 2; I_A:_ potassium current; I_K:_ delayed rectifier potassium current; Cav1.2: calcium channel, voltage-dependent, L type, alpha 1C subunit; Cav3.2: calcium channel, voltage-dependent, T type, alpha 1 H subunit; Ca^2+:^ calcium; K^+:^ potassium; BK_ca:_ calcium activated potassium current; ATP: adenosine tri-phosphate; cAMP:  cyclic adenosine mono-phosphate; LH: luteinizig hormone; I_h _: pacemaker current, hyperpolarization-activated current, hyperpolarization-activated cation current; RNA: ribonucleic acid; cDNA: complimentary deoxyribonucleic acid; SDS: sodium dodecyl sulfate; PMSF: phenylmethylsulfonyl fluoride; DCC: dicyclohexlcabodiimide; TTBS: tris buffered saline with 0.1% Tween 20; HRP: horseradish peroxidase; HCl: hydrochloric acid; PBS: phosphate buffered saline; hCG: human chorionic gonadotropin; FSH: follicle stimulating hormone; GnRH: gonadotropin releasing hormone; β: beta

## Competing interests

The authors declare that they have no competing interests.

## Authors' contributions

JY conceived of the study along with AA, and participated in its design and coordination and helped to draft the manuscript. BSK is responsible for the Western blots and RT-PCR, data and statistical analysis, and manuscript preparation. LG carried out the immunohistochemistry and baseline research articles for the initial research. JP carried out the immunohistochemistry, baseline research articles, data and statistical analysis, and manuscript preparation. CP carried out the immunohistochemistry as well as data analysis. AA conceived of the study along with JY, and participated in its design and coordination and helped to draft the manuscript. All authors read and approved the final manuscript.
